# Supplementary material for: Intracellular galectin-3 is a lipopolysaccharide sensor that promotes glycolysis through mTORC1 activation
Source: Nat Commun. 2022 Dec 8;13:7578. doi: 10.1038/s41467-022-35334-x (PMC9732310; doi:10.1038/s41467-022-35334-x)
Supplement: Supplementary file 1 — Supplementary Information [file 41467_2022_35334_MOESM1_ESM.pdf]

Supplementary Fig. 1

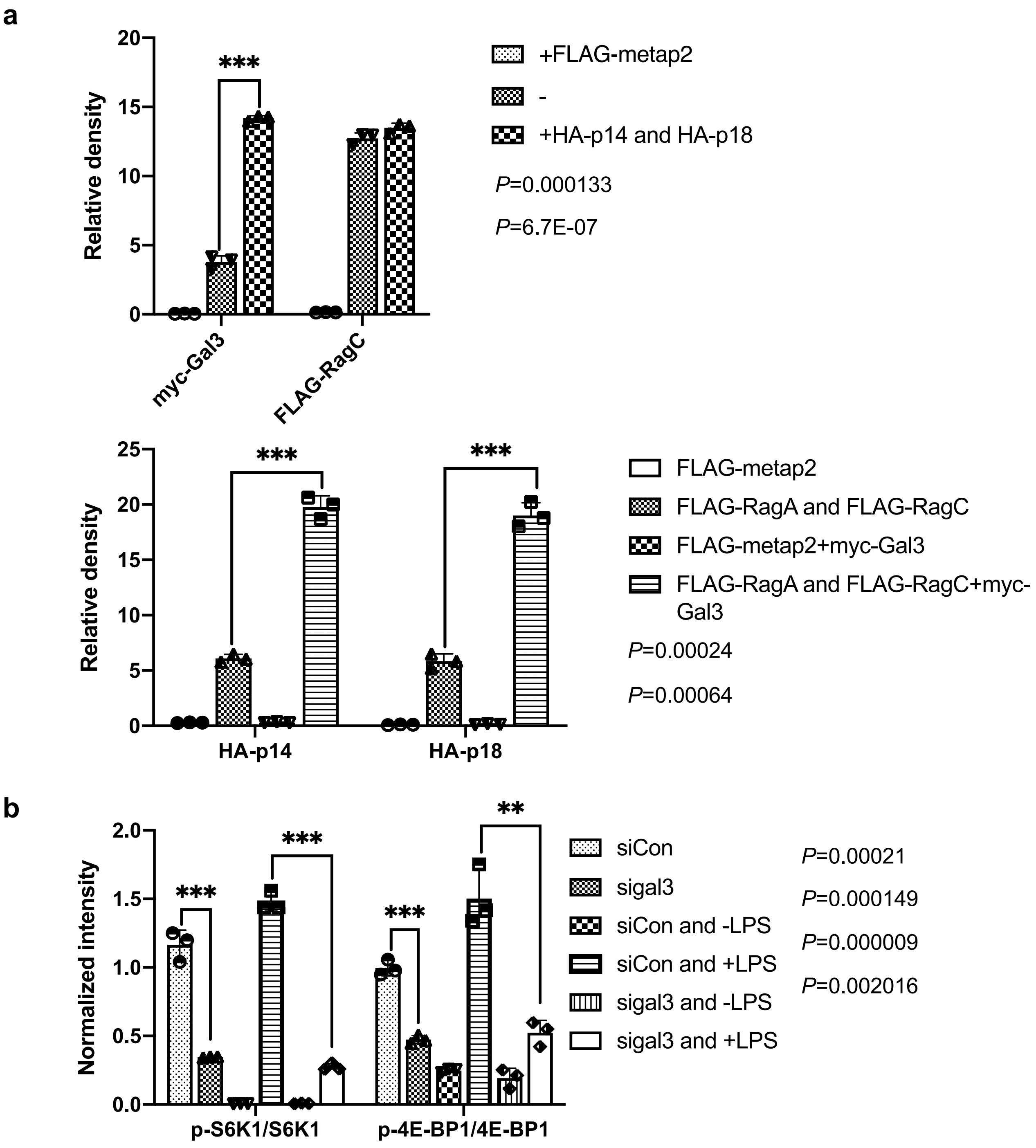

Supplementary Fig. 1. Galectin-3 facilitates the interaction of Rag GTPases and Ragulator to stimulate the mTORC1 signaling. a Quantitative analysis of the immunoblots in Fig. 2a. Each bar represents the mean ± SD for biological triplicate experiments. *P* values were calculated by two-tailed unpaired Student's *t* test (****P* < 0.001). **b** Quantitative analysis of the ratio of p-S6K1/S6K1 and p-4E-BP1/4E-BP1 in Fig. 2d. Error bars represent mean ± SD for biological triplicate experiments. *P* values were calculated by two-tailed unpaired Student's *t* test (***P* < 0.01, ****P* < 0.001).

Supplementary Fig. 2

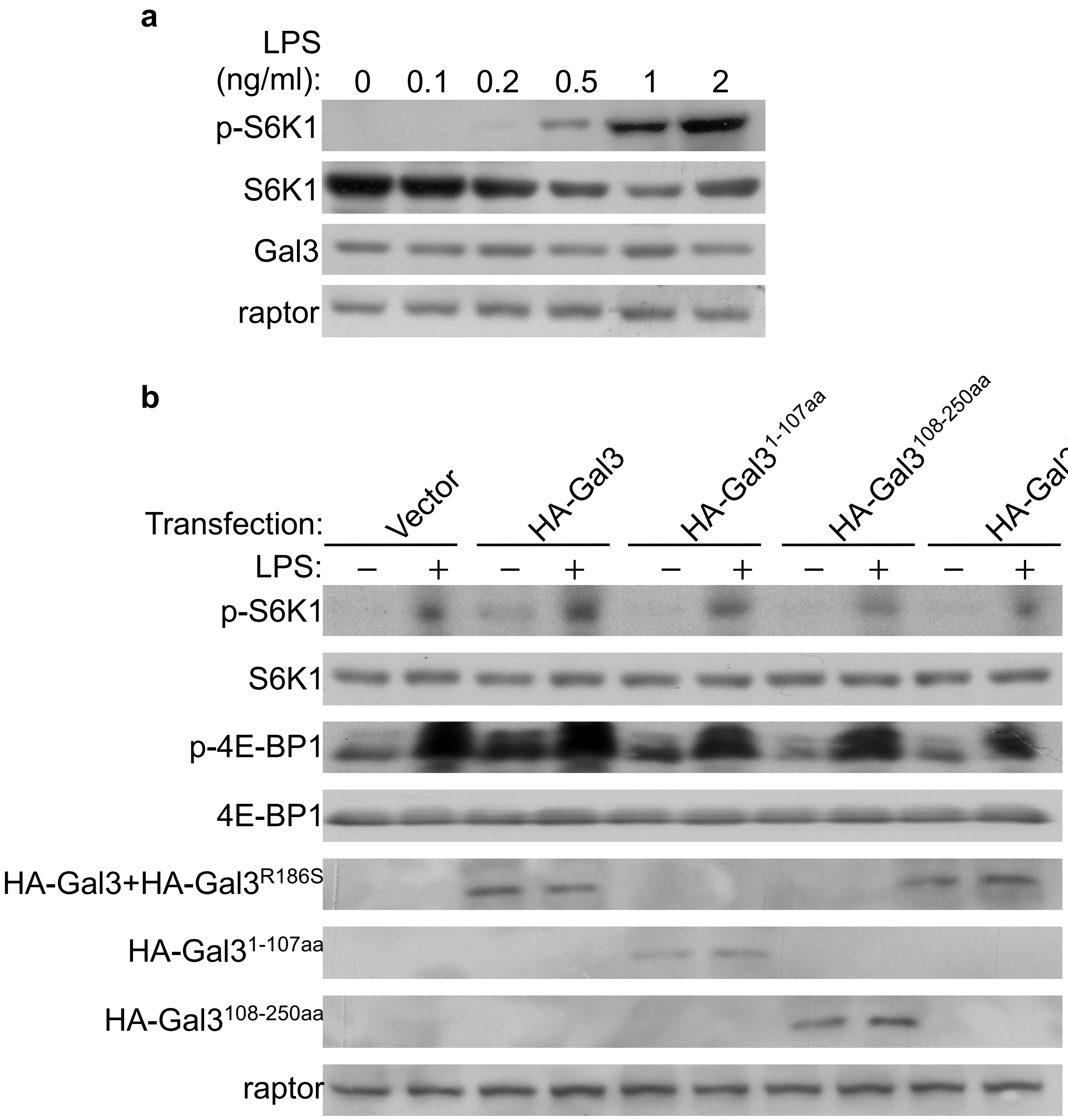

Supplementary Fig. 2. Galectin-3 senses LPS to activate mTORC1. a HEK-293T were treated with different concentrations of LPS for western blotting analysis of the expression or phosphorylation of the indicated proteins. Each experiment was repeated three times with similar results. **b** HEK-293T cells were transfected with HA-Gal3, HA-Gal3$^{1\text{-}107aa}$ (N-terminal domain), HA-Gal3$^{108\text{-}250aa}$ (C-terminal carbohydrate recognition domain), or HA-Gal3^{R186S}, starved of amino acids and glucose for 1 h, and stimulated with LPS (1 μg/ml) for 5 h for western blotting analysis of the expression or phosphorylation of the indicated proteins. Each experiment was repeated three times with similar results.

Supplementary Fig. 3

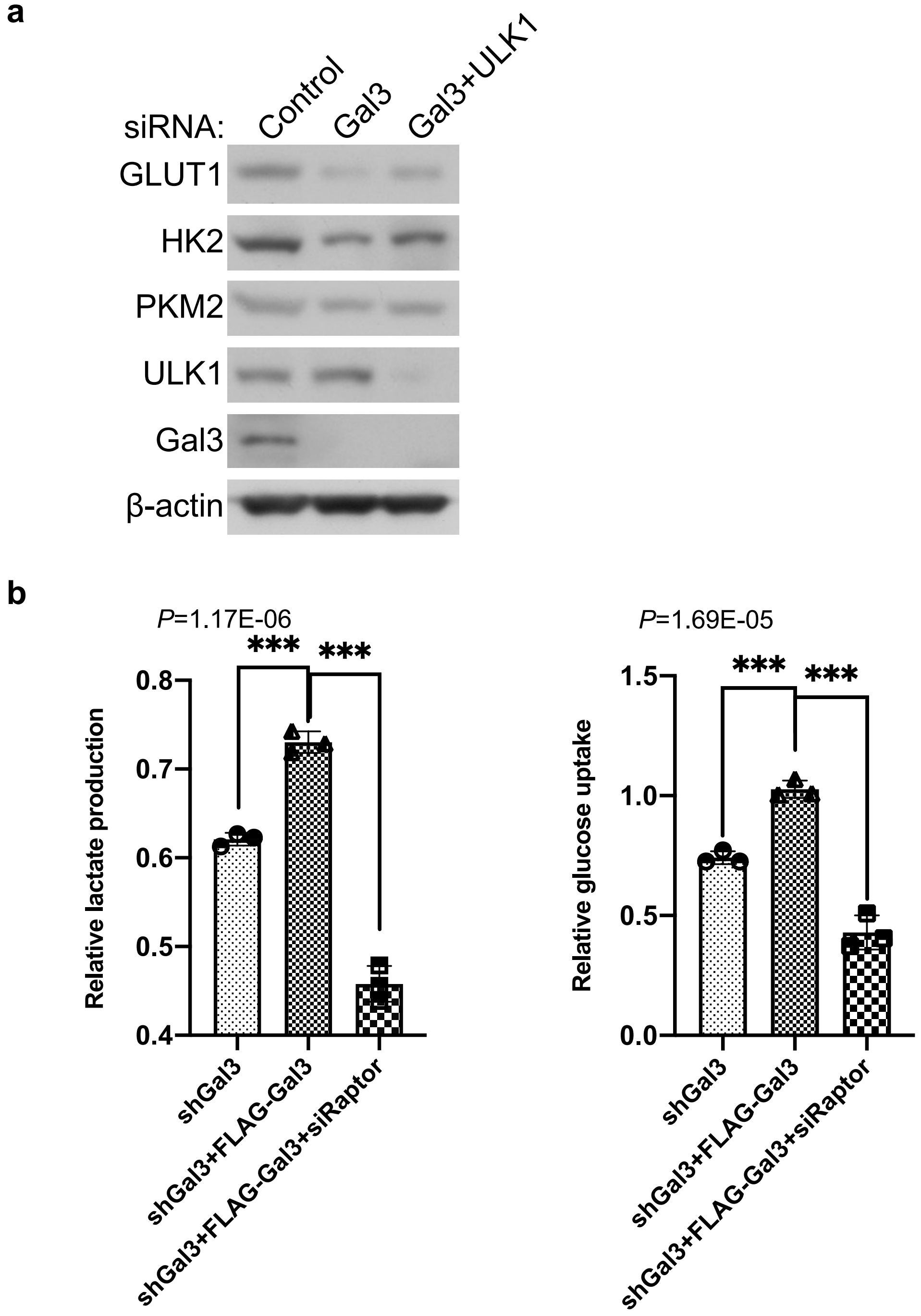

Supplementary Fig. 3. Galectin-3 promotes glycolysis through activating the mTORC1 signaling. a HEK-293T cells were treated with siCon, siGal3 or siGal3+siULK1 for western blotting analysis of the indicated proteins. Each experiment was repeated three times with similar results. **b** HEK-293T cells deficient of galectin-3 were transfected with FLAG-Gal3 or FLAG-Gal3+siRaptor for the measurement of glucose uptake and lactate production. Error bars represent mean ± SD for biological triplicate experiments. *P* values were calculated by one-way ANOVA (***$P < 0.001$).

Supplementary Fig. 4

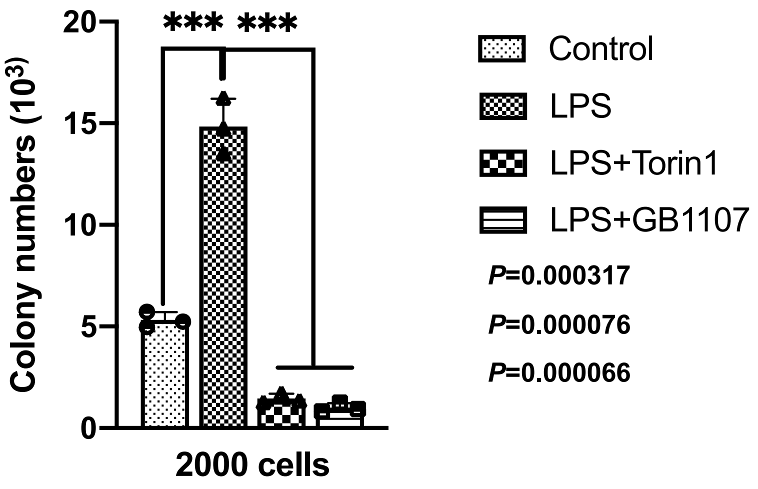

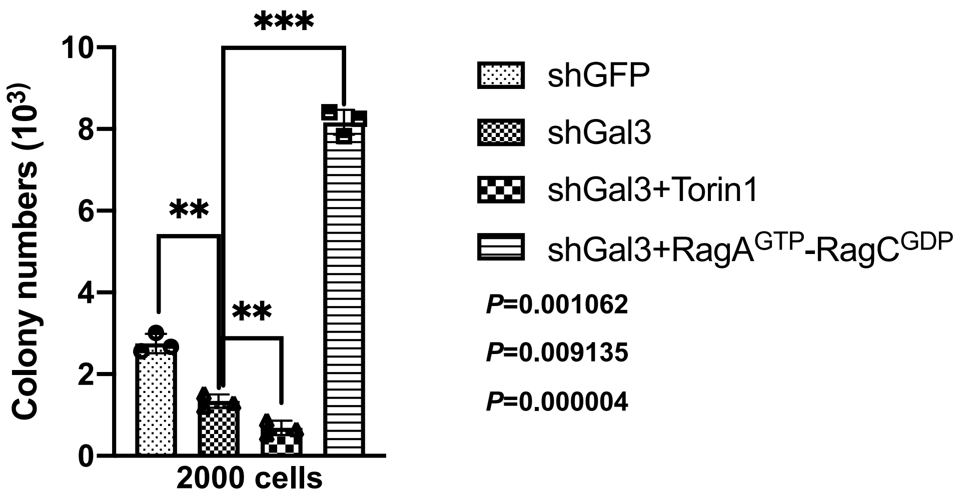

Supplementary Fig. 4. Galectin-3 is implicated in hepatocarcinogenesis. HepG2 cells were treated with LPS, LPS+Torin 1, or LPS+GB1107, or infected with lentiviruses carrying shGFP or shGal3 and transfected with FLAG-RagA^{GTP}/RagC^{GDP} in the presence or absence of Torin 1 and cultured for 10 days before staining with crystal violet for colony formation assays. The number of colony was counted. Error bars represent mean ± SD for biological triplicate experiments. *P* values were calculated by two-tailed unpaired Student's *t* test (***P* < 0.01, ****P* < 0.001).
