# Supplementary material for: Intracellular galectin-3 is a lipopolysaccharide sensor that promotes glycolysis through mTORC1 activation
Source: Nat Commun. 2022 Dec 8;13:7578. doi: 10.1038/s41467-022-35334-x (PMC9732310; doi:10.1038/s41467-022-35334-x)
Supplement: Supplementary file 3 — Description of Additional Supplementary Files [file 41467_2022_35334_MOESM3_ESM.pdf]

Description of Additional Supplementary Files

File Name: Supplementary Data 1
Description: Mass Spectrometry Analysis of galectin-3, RagA, or RagC-containing Protein Complex. Whole-cell extracts from HEK293T cells with expression of stably integrated FLAG-Gal3, FLAG-RagA, or FLAGRagC were purified with an anti-FLAG affinity column and analyzed by mass spectrometry. Information on Peptide fragments is shown.
