# Supplementary material for: Intracellular galectin-3 is a lipopolysaccharide sensor that promotes glycolysis through mTORC1 activation
Source: Nat Commun. 2022 Dec 8;13:7578. doi: 10.1038/s41467-022-35334-x (PMC9732310; doi:10.1038/s41467-022-35334-x)
Supplement: Supplementary file 5 — Reporting Summary [file 41467_2022_35334_MOESM5_ESM.pdf]

nature portfolio

Corresponding author(s): Yongfeng Shang
Last updated by author(s): Nov 21, 2022

Reporting Summary

Nature Portfolio wishes to improve the reproducibility of the work that we publish. This form provides structure for consistency and transparency in reporting. For further information on Nature Portfolio policies, see our Editorial Policies and the Editorial Policy Checklist.

Statistics

For all statistical analyses, confirm that the following items are present in the figure legend, table legend, main text, or Methods section.

n/a	Confirmed	
☐	☒	The exact sample size (*n*) for each experimental group/condition, given as a discrete number and unit of measurement
☐	☒	A statement on whether measurements were taken from distinct samples or whether the same sample was measured repeatedly
☐	☒	The statistical test(s) used AND whether they are one- or two-sided<br>*Only common tests should be described solely by name; describe more complex techniques in the Methods section.*
☒	☐	A description of all covariates tested
☐	☒	A description of any assumptions or corrections, such as tests of normality and adjustment for multiple comparisons
☐	☒	A full description of the statistical parameters including central tendency (e.g. means) or other basic estimates (e.g. regression coefficient) AND variation (e.g. standard deviation) or associated estimates of uncertainty (e.g. confidence intervals)
☐	☒	For null hypothesis testing, the test statistic (e.g. *F*, *t*, *r*) with confidence intervals, effect sizes, degrees of freedom and *P* value noted<br>*Give P values as exact values whenever suitable.*
☒	☐	For Bayesian analysis, information on the choice of priors and Markov chain Monte Carlo settings
☒	☐	For hierarchical and complex designs, identification of the appropriate level for tests and full reporting of outcomes
☐	☒	Estimates of effect sizes (e.g. Cohen's *d*, Pearson's *r*), indicating how they were calculated

Our web collection on statistics for biologists contains articles on many of the points above.

Software and code

Policy information about availability of computer code

Data collection	No software was used for data collection.
Data analysis	Graphpad Prism (Version 8)<br>Scipy1.8.0<br>DESeq2<br>Mascot software Revision 2.3.01

For manuscripts utilizing custom algorithms or software that are central to the research but not yet described in published literature, software must be made available to editors and reviewers. We strongly encourage code deposition in a community repository (e.g. GitHub). See the Nature Portfolio guidelines for submitting code & software for further information.

Data

Policy information about availability of data

All manuscripts must include a data availability statement. This statement should provide the following information, where applicable:

- Accession codes, unique identifiers, or web links for publicly available datasets
- A description of any restrictions on data availability
- For clinical datasets or third party data, please ensure that the statement adheres to our policy

The RNA-seq data generated in this study have been deposited in the NCBI Gene Expression Omnibus (GEO) under accession code GSE211784 [https://

www.ncbi.nlm.nih.gov/geo/query/acc.cgi?acc=GSE211784]. Mass spectrometry data are provided in Supplementary Data 1. Computational analysis of diabetes patient data from ArrayExpress (E-MEXP-2559) [https://www.ebi.ac.uk/biostudies/arrayexpress/studies/E-MEXP-2559]. Datasets of "thyroid gland papillary carcinoma", "prostatic intraepithelial neoplasia", and "hepatocellular carcinoma" from Oncomine database [https://www.oncomine.org]. The median/best cutoff of galectin-3 in TCGA liver cancer and related survival data were collected from Human Protein Atlas [https://www.proteinatlas.org/ENSG00000131981-LGALS3/pathology/liver+cancer]. All data generated or analyzed during this study are included in this published article (and its supplementary information files). The reporting summary and editorial checklist for this article are available as a Supplementary File. Source data are provided with this paper.

Human research participants

Policy information about studies involving human research participants and Sex and Gender in Research.

Reporting on sex and gender	n/a
Population characteristics	n/a
Recruitment	n/a
Ethics oversight	n/a

Note that full information on the approval of the study protocol must also be provided in the manuscript.

Field-specific reporting

Please select the one below that is the best fit for your research. If you are not sure, read the appropriate sections before making your selection.

☒ Life sciences ☐ Behavioural & social sciences ☐ Ecological, evolutionary & environmental sciences

For a reference copy of the document with all sections, see nature.com/documents/nr-reporting-summary-flat.pdf

Life sciences study design

All studies must disclose on these points even when the disclosure is negative.

Sample size	We did not perform sample size calculations and sample sizes were chosen according to accepted standards in the field. RNA sequencing was performed with 3 independent replicates as is typical for transcriptomic analysis of cell lines.
Data exclusions	No data was excluded from this study.
Replication	All experiments were repeated three times, unless indicated in the figure legend or methods. Small molecule library screening was performed in duplicate, all other experiments were performed in at least triplicate.
Randomization	All experiments were designed according to demand and did not involve randomization.
Blinding	The study did not involve blind selection. All experiments were designed according to the requirements and completed independently.

Reporting for specific materials, systems and methods

We require information from authors about some types of materials, experimental systems and methods used in many studies. Here, indicate whether each material, system or method listed is relevant to your study. If you are not sure if a list item applies to your research, read the appropriate section before selecting a response.

Materials & experimental systems

n/a	Involved in the study	
☐	☒	Antibodies
☐	☒	Eukaryotic cell lines
☒	☐	Palaeontology and archaeology
☒	☐	Animals and other organisms
☒	☐	Clinical data
☒	☐	Dual use research of concern

Methods

n/a	Involved in the study	
☒	☐	ChIP-seq
☒	☐	Flow cytometry
☒	☐	MRI-based neuroimaging

Antibodies

Antibodies used	αRagA (4357, 1:1000 for Western Blotting (WB)), αRagC (3360,1:1000 for WB and 1:100 for Immunofluorescence (IF)), αmTOR (2972 and 2983, 1:1000 for WB and 1:100 for IF), αraptor (2280, 1:500 for WB), α4E-BP1 (9644, 1:1000 for WB), αp-4E-BP1 (2855, 1:1000 for WB), αTSC2 (4308, 1:1000 for WB), αS6K1 (9202, 1:1000 for WB), αp-S6K1 (9205, 1:500 for WB), αNPRL2 (37344, 1:1000 for WB), αWDR59 (53385, 1:1000 for WB), αp18 (8975, 1:1000 for WB and 1:100 for IF), αp14 (8145, 1:1000 for WB), αMyc (2272, 1:5000 for WB), αFLAG (14793, 1:10000 for WB), αHK2 (2867, 1:1000 for WB), αGPI (94068, 1:1000 for WB), αLDHA (3582, 1:1000 for WB), αULK1 (8054, 1:1000 for WB), αPKM1 (7067, 1:1000 for WB), αPKM2 (4053, 1:1000 for WB), and Normal Rabbit IgG (2729, 1:100 for Immunoprecipitation) from Cell Signaling; αmTOR (ab32028, 1:100 for IF), αLamp2 (ab25631, 1:100 for IF), αATP6V1B2 (ab73404, 1:1000 for WB), αSLC38A9 (ab81687, 1:1000 for WB), αGalectin-3 (ab209344, 1:1000 for WB), αGalectin-4 (ab175185, 1:1000 for WB), αGalectin-2 (ab232703, 1:1000 for WB), αE. coli LPS (ab35654, 1:100 for IF), αGLUT1 (ab115730, 1:1000 for WB), and αPFKM (ab204131, 1:1000 for WB) from Abcam; αWDR24 (20778-1-AP, 1:1000 for WB) and αSEC13 (15397-1-AP, 1:1000 for WB) from Proteintech; αβ-actin (AC004, 1:10000 for WB), HRP Goat Anti-Rabbit IgG (AS014, 1:5000 for WB), and HRP Goat Anti-Mouse IgG (AS003, 1:5000 for WB) from ABclonal; αHA (YM3146, 1:5000 for WB) from Biomed; αDEPDC5 (LS-C256035, 1:100 for WB) from LSBio.
Validation	All antibodies are validated prior to use according to the validation claims on the manufacturer's website.

Eukaryotic cell lines

Policy information about cell lines and Sex and Gender in Research

Cell line source(s)	The cell lines (HEK-293T|HepG2|MEFs) used were obtained from the American Type Culture Collection (ATCC).
Authentication	no further authentication was performed for commercially available cell lines.
Mycoplasma contamination	All cell lines tested negative for Mycoplasma contamination.
Commonly misidentified lines (See ICLAC register)	No cell lines used are commonly misidentified.
